# Turner syndrome: counseling prior to oocyte donation

**DOI:** 10.1590/S1516-31802007000200009

**Published:** 2007-03-04

**Authors:** Ester Silveira Ramos

**Keywords:** Turner syndrome, Oocyte donation, Gonadoblastoma, Counseling, Y chromosome, Síndrome de Turner, Doação de oócitos, Gonadoblastoma, Aconselhamento, Cromossomo Y

## Abstract

Ovarian failure is a typical feature of Turner syndrome (TS). Patients are followed clinically with hormone replacement therapy (HRT) and inclusion in the oocyte donation program, if necessary. For patients with spontaneous puberty, genetic counseling regarding preimplantation genetic diagnosis and prenatal diagnosis is indicated. Patients with dysgenetic gonads and a Y chromosome are at increased risk of developing gonadoblastoma. Even though this is not an invasive tumor, its frequent association with other malignant forms justifies prophylactic gonadectomy. It is important to perform gonadectomy before HRT and pregnancy with oocyte donation. Among patients with TS stigmata and female genitalia, many have the Y chromosome in one of the cell lines. For this reason, all patients should undergo cytogenetic analysis. Nevertheless, in cases of structural chromosomal alterations or hidden mosaicism, the conventional cytogenetic techniques may be ineffective and molecular investigation is indicated. The author proposes a practical approach for investigating women with TS stigmata in whom identification of the X or Y chromosome is important for clinical management and follow-up.

## RESUMO

A falência ovariana é um achado típico da síndrome de Turner (ST). As pacientes podem ser submetidas à terapia de reposição hormonal (TRH) e incluídas em programas de doação de oócito, quando necessário. Para as pacientes com puberdade espontânea, está indicado o aconselhamento genético para a futura descendência abordando os diagnósticos genéticos pré-natal e pré-implantação. Pacientes com gônadas disgenéticas e cromossomo Y apresentam risco aumentado para desenvolvimento de gonadoblastoma. Embora esse tumor não seja invasivo, sua associação freqüente com tumores malignos justificaria a gonadectomia profilática. Entre as pacientes com estigmas da ST e genitália feminina, muitas apresentam cromossomo Y em pelo menos uma linhagem celular. Por essa razão, todas as pacientes devem ser submetidas à análise citogenética, para a realização de cirurgia antes do início da TRH e da gravidez com doação de oócito. No entanto, em casos de alteração cromossômica estrutural ou mosaicismo críptico, as técnicas citogenéticas convencionais podem não ser efetivas, estando indicada a investigação molecular. Uma abordagem prática para o médico investigar as pacientes com ST é proposta neste artigo, devido à importância da identificação do cromossomo Y ou de um segundo cromossomo X para o manejo clínico e o acompanhamento das pacientes.

The diagnosis of Turner syndrome (TS) is based on the characteristics described by Otto Ullrich and Henry Turner, such as short stature, gonadal dysgenesis (streak gonads), typical dysmorphic features, and abnormalities in organs such as the kidneys and heart. It may be defined as the combination of phenotypic features and complete or partial absence of one of the X chromosomes, frequently accompanied by cell line mosaicism.^1^

Ovarian failure is a typical feature of TS. Only 2% of these patients have natural pregnancies, with high rates of miscarriages, stillbirths and malformed babies. Therefore, hormone replacement therapy (HRT) is necessary to achieve the development of normal female sexual characteristics and to prevent cardiovascular complications and osteoporosis. Gonadal dysfunction among these women has been regarded as a major indication for oocyte donation. Their pregnancy rate in oocyte donation programs is 24-47%.^2^

The incidence of Y chromosome sequences in patients with TS stigmata has been evaluated in several studies. The introduction of molecular biology techniques such as the polymerase chain reaction (PCR) has revealed the existence of hidden mosaics not detected by cytogenetic examination, ranging in frequency from 0% to 61% according to the techniques used.^3,4^

The presence of Y chromosome material in individuals with the TS stigmata and gonadal dysgenesis is associated with the development of gonadoblastoma, a tumor containing nests of germ cells and cells resembling Sertoli or granulosa cells.^5^ The risk has been previously estimated to be up to 30%.^6^ The pathogenesis of gonadoblastomas and their malignant potential are still rather obscure, but the tumor is frequently associated with other malignant forms such as dysgerminomas. Gonadectomy is generally recommended; however, this consensus is questioned by more recent studies that have shown a risk of 7–10% for the development of gonadoblastoma, i.e. lower than the previously reported figures.^7,8^

Page hypothesized the existence of a gene on the Y chromosome (the *gonadoblastoma locus on the Y chromosome* or *GBY*).^9^ More recently, it has been speculated that more than one gene may be implicated in gonadoblastoma, and it is also possible that some relevant genes are present in multiple copies.^10^ The main candidate for *GBY* is *TSPY*, a gene that has several homologous copies. The *TSPY* genes are arranged in clusters on the Y chromosome and form part of the *TSPY*/*TSPY-like*/*SET*/*NAP-1* (TTSN) superfamily. *TSPY* expression is apparently restricted to male germ cells and their precursors, and begins during fetal development. The cellular site of expression suggests a function in spermatogonial proliferation.^11^ *TSPY* expression has been detected in gonadoblastoma tissues and recent studies have provided circumstantial evidence supporting a role for *TSPY* as an oncogene.^10,12,13^

The risk of gonadoblastoma is directly proportional to age. It is significantly higher after puberty and in patients with less virilized external genitalia (probably due to underlying dysgenetic structures).^6^ This might be explained by the Page hypothesis, which would suggest that the *GBY* has some physiological functions in normal testes but strongly predisposes dysgenetic gonads to develop gonadoblastomas, thus acting as an oncogene.

The Multidisciplinary Sex Determination and Differentiation Outpatient Service of the University Hospital of Faculdade de Medicina de Ribeirão Preto (FMRP), Universidade de São Paulo (USP), Brazil, which is a tertiary care hospital, receives many patients with TS stigmata. We propose here a protocol for investigating and following up patients with TS stigmata and female genitalia, based on the experience of our Service.

After determining the clinical diagnosis, all patients are given advice about the genetic tests. A multidisciplinary team provides pretest consultations covering: a) the genetic basis of TS; b) the necessity of cytogenetic and molecular analysis, and specific methodology; c) the benefits and risks of the genetic test; d) expectations regarding the test result; e) available options for prevention (prophylactic gonadectomy and clinical treatment), and the associated limitations and risks; f) assurance of confidentiality for all test results and related information; and g) psychological support. Written informed consent for testing is obtained from all patients or their legal guardians. The individuals are assessed periodically during the counseling sessions to verify that they understand the information.

One hundred metaphase spreads prepared from peripheral lymphocyte cultures are studied by conventional staining and GTG-banding techniques in order to detect mosaicism.^14,15^ CBG, G-11 banding, and fluorescent *in situ* hybridization (FISH) are used to identify the origin of ring and marker chromosomes.^15^

**Figure 1:**
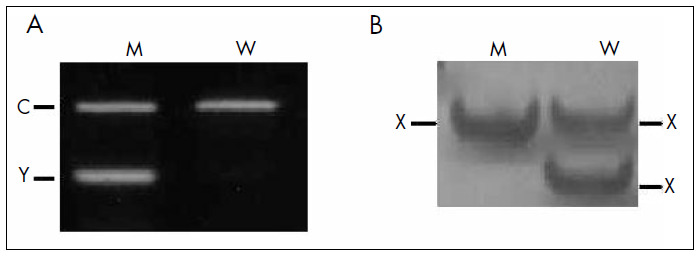
Results from electrophoresis in an agarose gel stained with ethidium bromide (A) and a polyacrylamide gel stained with silver nitrate (B). M = normal man; W = normal woman; C = amplification with the control primers (for example, sequence of the β-globin gene); Y = chromosome Y-specific sequence; X = chromosome X-specific polymorphic sequence.

In cases of structural chromosomal alterations or hidden mosaicism, the conventional cytogenetic techniques may be ineffective and molecular investigation is indicated. In these patients, DNA is extracted from peripheral blood leukocytes by standard methods and screened for the existence of Y chromosome material using PCR, with at least three different primer sets spanning the entire chromosome. For example, sequences of the genes *SRY* (*sex-determining region of the Y chromosome,* located on Yp short arm) (Figure 1), *TSPY* (pericentomeric region), and *DAZ* (*deleted in azoospermia*, located on Yq long arm).^16,17^ In cases with a 45,X karyotype, without mosaicism detected by cytogenetics analysis of 100 metaphases, we also collect buccal cells and urine, and perform nested PCR with the primers for *TSPY* sequences. Urine molecular analysis is a method of interest because it is an alternative to invasive procedures like skin biopsies and may be more informative about the gonadal cell lineage than blood or skin due to its embryological origin.^17^

To detect low-level mosaicism (blood and other tissues) involving a second X chromosome in 45,X patients, molecular analysis of PCR-based polymorphisms of X-specific genes may be used. For example, the human androgen receptor gene (*HUMARA)*, which is mapped on Xq12, has a polymorphic CAG repeat with 87% heterozygosity. In the presence of a second chromosome with a different *HUMARA* allele, the second band will be amplified by PCR (Figure 1).^18^

Considering the relationship between gonadoblastoma and a normal or anomalous Y chromosome in patients with TS stigmata, and the possibility of establishing the X or Y origin of chromosomal fragments, we use the following approach for patients with female external genitalia:

- *Group I — Patients with chromosome mosaicism*: (A) 45,X/46,XX (with normal or anomalous X): These patients are followed clinically with HRT and inclusion in the oocyte donation program, if necessary. For patients with spontaneous puberty, genetic counseling regarding the preimplantation genetic diagnosis and prenatal diagnosis is indicated. (B) 45,X/46,XY (with normal or anomalous Y): These patients are informed about the risk of gonadal neoplasia and the need for surgery, which is performed at the time of diagnosis. After gonadectomy, they undergo the same treatments and follow-up as patients with 45,X/46,XX. Detailed ultrasound examination of the gonads at regular intervals, or even magnetic resonance imaging, may be used to monitor some patients with Y chromosome material who do not accept surgery.
- *Group II — Patients with a 45,X karyotype and no mosaicism detected by cytogenetic and/or molecular techniques in multiple tissues*: these patients are followed clinically with HRT and inclusion in the oocyte donation program, but are monitored using ultrasound at six-month or one-year intervals, and in some cases using magnetic resonance imaging, to detect possible gonadal neoplasms that are not predicted by cytogenetic or molecular analysis. The determination of serum alphafetoprotein (AFP), beta-human chorionic gonadotropin (HCG), and/or other diagnostic markers may be used for additional data.^19,20^

For males with the 45,X/46,XY karyotype and normal external genitalia, the risk of developing gonadoblastoma is low.^21,22^ In our service, we perform an initial biopsy and annual clinical and ultrasound examinations of the gonads.

## CONCLUSIONS

It is important to perform gonadectomy in patients who are at risk of developing gonadoblastoma, before HRT and pregnancy by means of oocyte donation. On the other hand, most of the patients in the low-risk group, without the Y chromosome material, do not require gonadectomy and, in rare cases, could even have spontaneous puberty and fertility.

The University Hospital of FMRP has an oocyte donation program. Nevertheless, few data are available on the pregnancy rate and obstetric outcome following oocyte donation in TS patients.^23^

Clinical evaluation of the patients and laboratory identification of the normal or abnormal Y chromosome or a second X chromosome are important for the clinical management and prognostic counseling of patients with TS stigmata.

## References

[1] Ranke MB, Saenger P (2001). Turner's syndrome. Lancet.

[2] Abir R, Fisch B, Nahum R, Orvieto R, Nitke S, Ben Rafael Z (2001). Turner's syndrome and fertility: current status and possible putative prospects. Hum Reprod Update.

[3] Larsen T, Gravholt CH, Tillebeck A (1995). Parental origin of the X chromosome, X chromosome mosaicism and screening for "hidden" Y chromosome in 45,X Turner syndrome ascertained cytogenetically. Clin Genet.

[4] Coto E, Toral JF, Menendez MJ (1995). PCR-based study of the presence of Y-chromosome sequences in patients with Ullrich-Turner syndrome. Am J Med Genet.

[5] Jorgensen N, Muller J, Jaubert F, Clausen OP, Skakkebaek NE (1997). Heterogeneity of gonadoblastoma germ cells: similarities with immature germ cells, spermatogonia and testicular carcinoma in situ cells. Histopathology.

[6] Verp MS, Simpson JL (1987). Abnormal sexual differentiation and neoplasia. Cancer Genet Cytogenet.

[7] Hasle H, Olsen JH, Nielsen J, Hansen J, Friedrich U, Tommerup N (1996). Occurrence of cancer in women with Turner syndrome. Br J Cancer.

[8] Gravholt CH, Fedder J, Naeraa RW, Muller J (2000). Occurrence of gonadoblastoma in females with Turner syndrome and Y chromosome material: a population study. J Clin Endocrinol Metab.

[9] Page DC (1987). Hypothesis: a Y-chromosomal gene causes gonadoblastoma in dysgenetic gonads. Development.

[10] Tsuchiya K, Reijo R, Page DC, Disteche CM (1995). Gonadoblastoma: molecular definition of the susceptibility region on the Y chromosome. Am J Hum Genet.

[11] Vogel T, Schmidtke J (1998). Structure and function of TSPY, the Y-chromosome gene coding for the "testis-specific protein". Cytogenet Cell Genet.

[12] Lau YF (1999). Gonadoblastoma, testicular and prostate cancers, and the TSPY gene. Am J Hum Genet.

[13] Lau YF, Lau HW, Komuves LG (2003). Expression pattern of a gonadoblastoma candidate gene suggests a role of the Y chromosome in prostate cancer. Cytogenet Genome Res.

[14] Hook EB (1977). Exclusion of chromosomal mosaicism: tables of 90%, 95% and 99% confidence limit and comments on use. Am J Hum Genet.

[15] Verma R, Babu A (1995). Human chromosomes: principles and techniques.

[16] Ramos ES, Moreira CA, Vicente YA (1996). SRY-negative true hermaphrodites and an XX male in two generations of the same family. Hum Genet.

[17] Bartmann AK, Ramos ES, Caetano LC, Rios AF, Vila RA (2004). TSPY detection in blood, buccal, and urine cells of patients with 45,X karyotype. Am J Med Genet A.

[18] Yorifuji T, Muroi J, Kawai M, Sasaki H, Momoi T, Furusho K (1997). PCR-based detection of mosaicism in Turner syndrome patients. Hum Genet.

[19] Talerman A, Haije WG, Baggerman L (1978). Serum alphafetoprotein (AFP) in diagnosis and management of endodermal sinus (yolk sac) tumor and mixed germ cell tumor of the ovary. Cancer.

[20] Schanne FJ, Cooper CS, Canning DA (1999). False-positive pregnancy test associated with gonadoblastoma. Urology.

[21] Muller J, Ritzen EM, Ivarsson SA, Rajpert-De Meyts E, Norjavaara E, Skakkebaek NE (1999). Management of males with 45,X/46,XY gonadal dysgenesis. Horm Res.

[22] Slowikowska-Hilczer J, Romer TE, Kula K (2003). Neoplastic potential of germ cells in relation to disturbances of gonadal organogenesis and changes in karyotype. J Androl.

[23] Bodri D, Vernaeve V, Figueras F, Vidal R, Guillen JJ, Coll O (2006). Oocyte donation in patients with Turner's syndrome: a successful technique but with an accompanying high risk of hypertensive disorders during pregnancy. Hum Reprod.

